# Core-shell Ni/SiO_2_@ZrO_2_ catalyst for highly selective CO_2_ conversion accompanied by enhancing reaction stability

**DOI:** 10.1016/j.heliyon.2024.e40697

**Published:** 2024-11-27

**Authors:** Sha Cui, Zhe Wang, Honggang Zhao, Houxiang Sun, Qinhong Wei, Luhui Wang

**Affiliations:** a School of Petrochemical Engineering & Environment, Zhejiang Ocean University, Zhoushan, 316022, China; b School of Biology and Chemical Engineering, Panzhihua University, Panzhihua, 617000, China; c Zhejiang Provincial Key Laboratory of Petrochemical Pollution Control, Zhejiang Ocean University, Zhoushan, 316022, China; d National-Local Joint Engineering Laboratory of Harbor Oil & Gas Storage and Transportation Technology, Zhejiang Ocean University, Zhoushan, 316022, Zhejiang, China

**Keywords:** Core-shell structure, ZrO_2_, In-situ hydrothermal synthesis, CO_2_ RWGS

## Abstract

CO_2_ RWGS reaction was considered to be a promising process for carbon dioxide conversion, however it retained a big challenge owing to methanation and metal sintering. Therefore, it was desperately needed to devise highly selective and stable catalyst. Herein, core-shell Ni/SiO_2_@ZrO_2_ catalyst was successfully prepared via a combination of the wet impregnation and in-situ hydrothermal synthesis method, with ZrO_2_ as the coating shell. The optimized Ni/SiO_2_@4ZrO_2_ catalyst possessed enhanced metal-support interaction and rich oxygen vacancies as well as abundant medium-strength CO_2_ adsorption sites. As a result, under the GHSV of 120000 mL/g·h and 150000 mL/g·h, Ni/SiO_2_@4ZrO_2_ displayed a considerable hydrogenation activity and significantly higher selectivity to CO, compared with the Ni/SiO_2_ catalyst as a reference. During stability tests, Ni/SiO_2_@4ZrO_2_ also showed a superior catalytic stability with a steady 100 % CO selectivity, carried out at 600 °C for 72 h. This work provided a novel strategy of designing a core-shell catalyst for CO_2_ RWGS reaction, and was expected to be put into use in other multiphase reaction process.

## 1 Introduction

As global warming and climate change became increasingly problematic, the reduction strategies of greenhouse gas emission had attracted wide attention in terms of basic research and industrial applications. Carbon dioxide was the major contributor to the greenhouse effect and greatly increased due to the excessive consumption of coal, oil and natural gas [1]. Therefore, it was an urgent and common goal pursued by the international community to reduce carbon dioxide emissions [2,3]. In this case, applying CO_2_ as an alternative carbon source and converting it into high value-added chemicals and fuels satisfied the need to achieve large-scale carbon fixation, carbon emission reduction and carbon cycling. The use of blue and green hydrogen (H_2_) from renewable sources to react with CO_2_ to produce CO (RWGS, Eq. (1)) was a sustainable development direction that effectively utilized this abundant and relatively cheap carbon resource [4]. And the produced CO or syngas (CO + H_2_) was a key chemical platform molecule for conversion into other high value-added chemicals, such as methanol, high-carbon alcohols and other liquid fuels [5,6]. Moreover, most syngas conversion was relatively mature in commercial technology and could play a huge use of CO [7]. However, due to the thermodynamic stability of CO_2_, the numerous parallel reactions in the CO_2_ hydrogenation process and uncontrollable C-C coupling steps, it was difficult to balance the activity and selectivity [8]. Therefore, it was the core issue to achieve efficient and selective conversion of CO_2_ to CO for RWGS reaction, and the key was the development of catalyst.

(1) $$CO_{2}+H_{2}\to CO+H_{2}O,\Delta H_{298K}^{0}=+41.5kJ/mol$$

(2) CO2+4H2→CH+42H2O,ΔH298K0=−165kJ/mol

(3) $$CO+3H_{2}\to CH_{4}+H_{2}O,\Delta H_{298K}^{0}=-206.1kJ/mol$$

Up to now, numerous catalysts had been studied for RWGS reaction to tend to produce CO, including noble metal as Pt-, Au- and Ir-based catalysts [[9], [10], [11], [12]], and transition metal as Ni- and Cu-based catalysts [[13], [14], [15]]. The endothermic RWGS reaction required a higher reaction temperature, which often gave rise to agglomerating and sintering of metal catalysts, especially Cu-based catalysts. Considering economic benefits and natural reserves, noble metals could not be widely exploited. Ni-based catalyst had been widely used in RWGS reaction because of its economic viability and relatively high hydrogenation ability. However, the process of hydrogenation reduction of carbon dioxide to carbon monoxide was often accompanied by severe methanation reaction (Eq. (2) and Eq. (3)), and its competitive effect had a great influence on the selectivity of RWGS products. In fact, methane was the world's second largest greenhouse gas after carbon dioxide [16], and CH_4_ was a very stable molecule, and its further conversion was much more difficult than CO [17]. In order to improve the CO selectivity and stability, many researchers had explored a variety of modification strategies and methods for Ni-based catalysts. Considering the effective promotion to CO formation, reducing the size of nickel nanoparticles was frequently used. Wu et al. [18] employed Ni/SiO_2_ with 0.5 wt% and 10 wt% Ni loading for catalytic CO_2_ hydrogenation reaction. The 0.5 wt% Ni/SiO_2_ catalyst with small Ni particle size showed a comparatively higher CO selectivity, however the 10 wt% Ni/SiO_2_ catalyst with large Ni grain size and a low dispersion was inclined to favor CH_4_ formation. Goncalves et al. [19] prepared the Ni/SiO_2_ catalyst via a magnetron sputtering deposition method, and the catalyst exhibited small grain size, thus a high CO selectivity. Ni-based bimetallic catalysts have been widely studied for enhancing RWGS reaction performance. Zhu and coworkers [20] proposed the deposition of Ag on the surface of Ni-based catalyst, where Ag improved the properties of Ni through geometric and electronic effects which facilitated CO desorption and blocked undesirable methanation reaction. Yang et al. [21] found that Ni-FeO_X_/CeO_2_-Al_2_O_3_ exhibited the superior catalyst performance in the reaction of RWGS than Ni/CeO_2_-Al_2_O_3_ as a reference catalyst, not only in terms of activity, but also in terms of stability and selectivity towards CO, on account of the strong interaction between FeO_X_ and Ni particles. Beyond that, regulating the metal-support interaction (MSI) was an important strategy to promote the catalytic activity and improve the stability of supported metal nanoparticles. Rutherford et al. [22] compared Ni-based catalysts supported on Al_2_O_3_, ZnO, Fe_2_O_3_ and Co_3_O_4_ for RWGS reaction, and discovered that Ni/Co_3_O_4_ displayed upper CO selectivity which was realized by the metal-support interaction (MSI) between Ni and Co_3_O_4_ that suppressed CH_4_ formation. Wang et al. [23] found that the strong metal-support interactions between Ni and ZnO showed a remarkable positive effect on the CO selectivity, the light-degree MSI state however facilitated promotion of the CH_4_ selectivity and stability of methanation.

As was well-known that the support acted a pivotal role in supported Ni-based catalysts, reflected in facilitating Ni dispersion, modifying Ni electronic properties, introducing oxygen vacancy and mobility [[24], [25], [26]]. Various supports have been investigated for Ni-based catalysts, such as SiO_2_ [27,28], Al_2_O_3_ [29,30], CeO_2_ [24,31], TiO_2_ [32,33], ZnO [34] and ZrO_2_ [[35], [36], [37]]. SiO_2_ was widely used as a carrier for various heterogeneous catalysts because of its rich content, low cost and high specific surface area. Traditional silica had long been considered as an inert carrier for dispersing active metals or for exploring reaction mechanisms of heterogeneous catalysis [38]. ZrO_2_ was a P-type semiconductor and showed superior speciality as a reducible support or promoter for CO_2_ hydrogenation reaction, could provide abundant oxygen vacancies that facilitated the activation and reduction of CO_2_ [39]. Moreover, ZrO_2_ had hydrophilic characteristics that was conducive to the desorption of produced water [40,41], and this would definitely facilitate the RWGS reaction.

After considering the strategies above, in our work, Ni/SiO_2_ was first prepared by wet impregnating method. Then the as-synthesized Ni/SiO_2_ was used as the core material for coating with ZrO_2_ shell via in-situ hydrothermal process. And the core-shell Ni/SiO_2_@ZrO_2_ materials composed of Ni/SiO_2_ core and ZrO_2_ shell was prepared. The permeable porous shell of the core-shell catalyst protected metal particles from agglomeration and sintering [42,43], or provide a controlled nanopore environment facilitating the hydrogenation of CO_2_ to CO [44]. The formed Ni/SiO_2_@ZrO_2_ catalyst was tested for RWGS reaction at 400–600 °C, and Ni/SiO_2_ was used as a comparison. The CO_2_ conversion and CO selectivity was tested in order to evaluate the activity and stability of catalyst for RWGS reaction.

## 2 Experimental

### 2.1 Catalyst preparation

The core-shell Ni/SiO_2_@ZrO_2_ catalyst was prepared via a combination of the wet impregnation and in-situ hydrothermal synthesis method. First, Ni/SiO_2_ was prepared by a wet saturated volume impregnation method. In detail, 0.55 g of nickel nitrate (Ni(NO_3_)_2_·6H_2_O) was added to about 2 mL of deionized water (determined by the water absorption of SiO_2_). After forming a uniform solution, 1 g of SiO_2_ support was added to above solution and left for 12 h. Afterwards the sample was dried at 100 °C overnight, calcined at 600 °C for 3 h and reduced in hydrogen in-situ at 600 °C before reaction. The nickel weight percentage was calculated 10 wt%. Then, the above calcined Ni/SiO_2_ powder was dispersed in 50 mL of ethanol in vibration, followed by adding 2 mL and 4 mL ZrOCl_2_ solution (Zr molar concentration of 1 mol/L). 28 wt% ammonia solution was then added dropwise to the above solution for regulating pH ~ 9, then further stirred at room temperature for 2 h. The resultant suspension was further transferred into autoclaves for hydrothermal reaction at 150 °C for 12 h. Subsequently, the initially obtained Ni/SiO_2_@ZrO_2_ was collected by centrifugation and washing repeatedly. The collected sample was dried, calcined and reduced under the same conditions. The catalysts were denoted to Ni/SiO_2_@2ZrO_2_ and Ni/SiO_2_@4ZrO_2_, respectively.

### 2.2 Catalyst characterization

X-Ray Diffraction (XRD) was used for the crystalline structures of Ni/SiO_2_, Ni/SiO_2_@2ZrO_2_ and Ni/SiO_2_@4ZrO_2_ catalysts and the XRD patterns were obtained on a Rigaku SmartLab SE X-ray Diffractometer. The samples were measured from 10° to 80° at a scanning rate of 2°/min.

The elemental composition of catalysts was determined by ICP-OES and measured by Agilent 720-ES.

H_2_-TPR and CO_2_-TPD measurements were carried out by a PCA-1200 analyzer. The H_2_-TPR experiment was carried out to illustrate the reducibility of nickel catalysts and the CO_2_-TPD test was performed to obtain the information of the type and number of CO_2_ adsorption active sites. Prior to the experiments, 100 mg of the catalyst precursor was pretreated in Ar gas at 200 °C to remove the impurity adsorbed on the surface. Then for the H_2_-TPR, the calcined catalysts of Ni/SiO_2_, Ni/SiO_2_@2ZrO_2_ and Ni/SiO_2_@4ZrO_2_ were reduced in H_2_/Ar (10 % H_2_) flow of 40 mL/min from 20 °C to 890 °C at a heating rate of 5 °C/min and so the H_2_-TPR curve was recorded. And for the CO_2_-TPD, the pretreated catalyst precursor was firstly reduced by H_2_ at 600 °C for 2 h and subsequently cooled down to 100 °C. Then after an adsorption of CO_2_ gas for 1 h, the sample was purged with a Ar flow (20 mL/min) for 10 min. Afterwards, CO_2_ desorption was proceeded from 100 °C to 700 °C in Ar flow at a heating rate of 5 °C/min and the desorbed CO_2_ was detected by TCD.

X-ray photoelectron spectroscopy (XPS) was used to obtain information about the composition, chemical state and molecular structure of the elements on the surface of the sample. And the XPS spectra of the freshly reduced Ni/SiO_2_, Ni/SiO_2_@2ZrO_2_ and Ni/SiO_2_@4ZrO_2_ were acquired with an American Thermo Scientific K-Alpha spectrometer equipped with a Al-Ka source and an optimum analytical vacuum of 5.0 × 10^−7^ mbar. The binding energies (BE) was obtained with C1s = 284.80 eV as the standard.

Transmission electron microscopy (TEM) was measured to observe the morphology of the sample, the dispersion of the metal active phase and the particle size distribution. And the TEM photograph was determined with a FEI Tecnai G2 F20 electronic microscope. The interplanar distance was analyzed by Gatan DigitalMicrograph software and particle size distribution was calculated by Nano Measure 1.2 software.

### 2.3 Catalyst tests

The CO_2_ hydrogenation activity evaluation was executed in a fixed bed reactor equipped with a 8 mm quartz tube. A certain amount of catalyst was evenly mixed with quartz sand and then loaded in the middle of the quartz tube. The catalyst filling amount was 40 mg and 50 mg, and the corresponding weight-hour space speed was 150000 mL/g·h and 120000 mL/g·h. Before the reaction, the catalyst precursor was reduced in-situ with 20 % H_2_/N_2_ atmosphere from room temperature to 600 °C and then maintained for 1 h. Subsequently, the reactor was cooled to 400 °C and then introduced of reactive gas composed of 40 mL/min CO_2_, 40 mL/min H_2_ and 20 mL/min N_2_. The reaction was carried out under atmospheric pressure. The reaction temperature was set to increase from 400 °C to 600 °C, and then drop to 400 °C with the temperature interval of 50 °C. Each reaction temperature was kept for 1 h, and chromatographic analysis was performed four times. The reaction data were calculated by averaging the four test data. The conversion (X) and reaction rates (R) of CO_2_, as well as CO selectivity (Y_CO_) were calculated according to the following formula:

$$X_{CO_{2}}=\frac{{\left[CO_{2}\right]}_{\text{in}}-{\left[CO_{2}\right]}_{\text{out}}}{{\left[CO_{2}\right]}_{\text{in}}}\times 100\%$$

$$R=\frac{{\left[CO_{2}\right]}_{\text{in}}*X_{CO_{2}}}{m*w_{Ni}}\times 100\%$$

$$Y_{CO}=\frac{{\left[CO\right]}_{\text{out}}}{{\left[CO\right]}_{\text{out}}+{\left[CH_{4}\right]}_{\text{out}}}\times 100\%$$

where m standed for the loading mass of catalyst, ω_Ni_ was the mass fraction of nickel.

## 3 Results and discussion

### 3.1 Characterization results

**Fig. 1:**
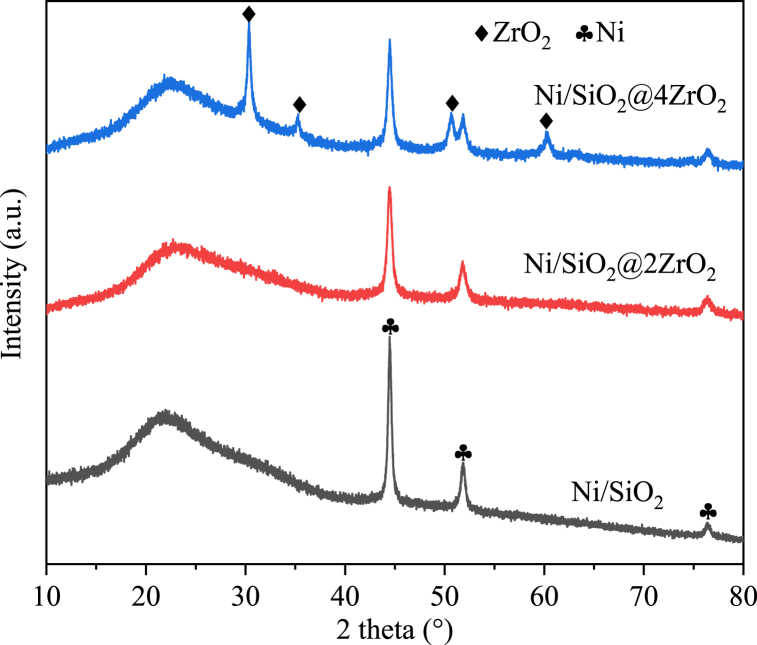
XRD patterns of reduced Ni/SiO_2_, Ni/SiO_2_@2ZrO_2_ and Ni/SiO_2_@4ZrO_2_ samples.

As determined in Table 1 by ICP-OES, Ni loading was 9.3 wt% in Ni/SiO_2_ close to the calculative 10 wt%. After coating with ZrO_2_, a normal nickel loading was 7.9 wt% and 7.6 wt % for Ni/SiO_2_@2ZrO_2_ and Ni/SiO_2_@4ZrO_2_. To identify the crystallographic structure of synthesized materials, the power X-ray diffraction study was employed and illustrated in Fig. 1. All catalysts displayed a typical broad peak at 22° that was owing to amorphous silica spheres [45]. The reduced Ni/SiO_2_ catalyst showed three shark peaks concentrated upon 44.5°, 52.0° and 76.5°, which were assigned to (111), (200), and (220) plane of cubic metallic Ni phase, respectively, according to JCPDS 04–0850. Whereas the overt decrease in the intensity of Ni peaks was appeared in Ni/SiO_2_@2ZrO_2_ and Ni/SiO_2_@4ZrO_2_, which was due to the coating with ZrO_2_. Beyond that Ni/SiO_2_@4ZrO_2_ also exhibited additional diffraction peaks at 30.3°, 35.4°, 50.4° and 59.5°, which were ascribed to tetragonal ZrO_2_ crystal phase (JCPDS 96-210-0390). It manifested that ZrO_2_ had been successfully prepared. However, Ni/SiO_2_@2ZrO_2_ exhibited no the characteristic peaks of zirconium, probably because less zirconium was uniformly distributed in the catalyst.

**Table 1:** Element composition and properties of samples.

Catalysts	Ni loading (wt.%)^a^	Bulk Ni/Zr (molar ratio)^a^	Surface Ni/Zr (molar ratio)^b^	Zr^δ+^/(Zr^δ+^+Zr^4+^)^b^	O_β_/(O_α_+O_β_)^b^
Ni/SiO_2_	9.3	–	–	–	–
Ni/SiO_2_@2ZrO_2_	7.9	1.64	0.55	0.24	0.35
Ni/SiO_2_@4ZrO_2_	7.6	1.32	0.20	0.36	0.60

a Ni content in the catalysts was analyzed by ICP-OES.

b Measured by XPS.

**Fig. 2:**
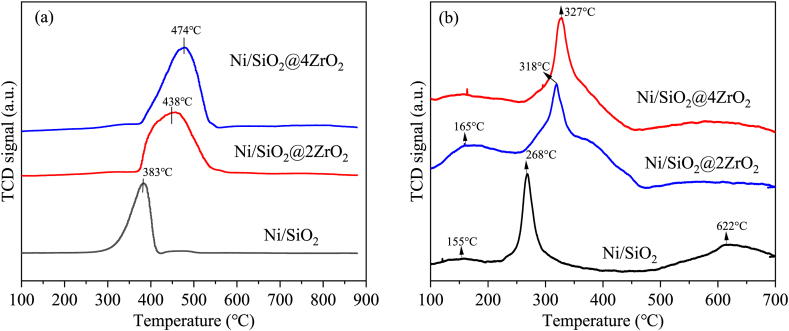
(a) H_2_-TPR of calcined Ni/SiO_2_, Ni/SiO_2_@2ZrO_2_ and Ni/SiO_2_@4ZrO_2_, (b) CO_2_-TPD of reduced Ni/SiO_2_, Ni/SiO_2_@2ZrO_2_ and Ni/SiO_2_@4ZrO_2_.

The reduction behavior of the calcined Ni/SiO_2_, Ni/SiO_2_@2ZrO_2_ and Ni/SiO_2_@4ZrO_2_ was investigated using H_2_-TPR experiments, as shown in Fig. 2a. The reduction temperature of NiO species was linked with the interaction strength of Ni species with the support and the higher was the reduction temperature, the greater was the metal-support interaction strength [46,47]. Only one reduction peak was observed for three samples that was linked to the reduction of the whole Ni^2+^ species. The reduction temperature of Ni/SiO_2_ was positioned at 383 °C, however that of Ni/SiO_2_@2ZrO_2_ and Ni/SiO_2_@4ZrO_2_ centered at 438 °C and 474 °C, respectively. Therefore, after coating with ZrO_2_, there was stronger interaction between surface NiO and support which could stabilize Ni species at high temperature. The amount of H_2_ consumption for Ni/SiO_2_ was about 1.19 mmol/g_cat_, which was slightly lower than the theoretical value of 1.20 mmol/g_cat_. On the contrary, H_2_ consumption was approximately 1.38 mmol/g_cat_ for Ni/SiO_2_@4ZrO_2_, which was greater than the theoretical value. This indicated that the lattice oxygen on zirconia surface was removable, whereas that on silica surface could not mobilized.

For the sake of comparing the CO_2_ adsorption capacity and accessibility of Ni/SiO_2_, Ni/SiO_2_@2ZrO_2_ and Ni/SiO_2_@4ZrO_2_, CO_2_-TPD was performed and the results were displayed in Fig. 2b. Typically, according to the desorption temperature, the desorption peak lower than 200 °C was ascribed to CO_2_ adsorption on the weak basic sites, and the desorption peak centered at 200–400 °C was attributed to intermediate adsorption basic sites, while the desorption peak higher than 400 °C was due to strongly chemisorbed CO_2_ sites [48]. Ni/SiO_2_ exhibited the weak (155 °C), intermediate (268 °C) and strong (622 °C) types of CO_2_ adsorption sites. However, Ni/SiO_2_@2ZrO_2_ showed simply two peaks, one smaller peak of the representative weak (165 °C) types of CO_2_ adsorption sites and another prominent peak (318 °C) of typical intermediate CO_2_ adsorption sites. When coating with more ZrO_2_, there was only one outstanding adsorption peak centered around 327 °C present to Ni/SiO_2_@4ZrO_2_. It suggested that the introduced ZrO_2_ to the catalyst surface could improve the adsorption performance for CO_2_ molecules which was attributed to the increase in intermediate adsorption basic sites. As manifested in the literature [49], the Lewis basicity of O^2−^ vacancies on the surface of ZrO_2_ may make them alkaline sites for CO_2_ adsorption. Hence, detailed comparing Ni/SiO_2_ and Ni/SiO_2_@4ZrO_2_, apart from the intermediate adsorption for CO_2_ active sites, Ni/SiO_2_ also furnished weak and strong adsorption active sites for CO_2_ element. But as we know, during the reaction, the weakly adsorbed carbon dioxide could be not broken of the chemical bonds between C-O, and desorption from the active sites became very difficult owing to the strongly adsorbed sites. Therefore, it was not conducive to the CO_2_ conversion reaction for extremely weak or fairly strong adsorption of carbon dioxide to the catalyst surface. It was the medium alkaline site that acted an important part in the adsorption and activation of carbon dioxide [50], due to the formation of many intermediates, such as HCO_3_^−^, b-CO_3_^2-^, m-CO_3_^2-^ or carboxylate on this site. In summary, Ni/SiO_2_@4ZrO_2_ showed better carbon dioxide adsorption performance for CO_2_ hydrogenation.

**Fig. 3:**
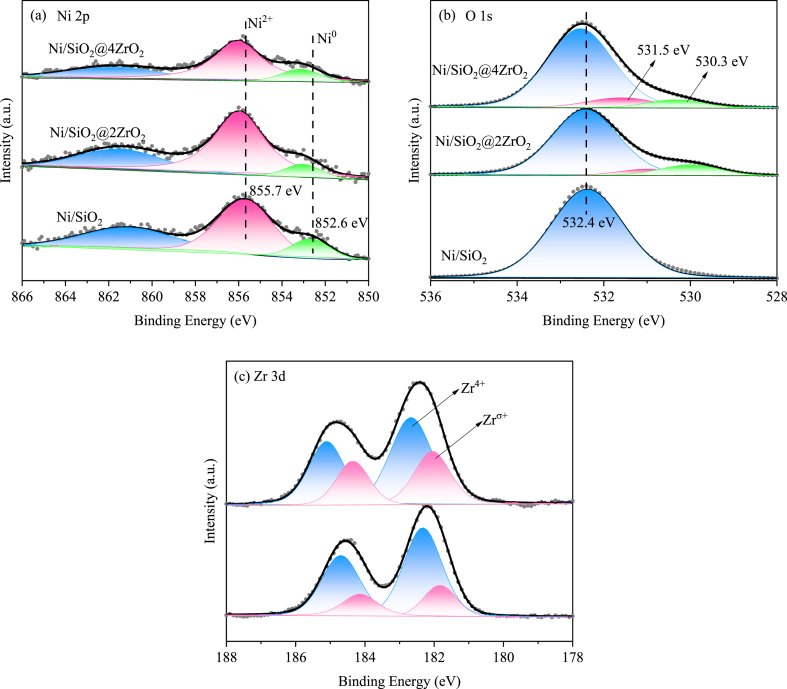
XPS profiles of (a) Ni 2p, (b) O 1s, (c) Zr 3d in the reduced Ni/SiO_2_, Ni/SiO_2_@2ZrO_2_ and Ni/SiO_2_@4ZrO_2_ catalysts.

The surface chemical state of reduced Ni/SiO_2_, Ni/SiO_2_@2ZrO_2_ and Ni/SiO_2_@4ZrO_2_ were showed in the XPS results. In spectra of Ni 2p (Fig. 3a) all samples displayed three fitted peaks, one peak appeared at about 852.6 eV was assigned to Ni^0^ and another peak centered at approximately 855.7 eV with a satellite peak signal centered at 861.0 eV was attributed to Ni^2+^ in the NiO phase [51,52]. Further observation revealed that after coating with ZrO_2_, the binding energy of Ni^0^ and Ni^2+^ over Ni/SiO_2_@2ZrO_2_ and Ni/SiO_2_@4ZrO_2_ both shifted to the higher binding energy by 0.4 and 0.6 eV respectively, indicated a stronger interaction between the Ni species and support. The O 1s XPS spectra was depicted in Fig. 3b. For Ni/SiO_2_@2ZrO_2_ and Ni/SiO_2_@4ZrO_2_, the O 1s XPS spectra were deconvoluted into three main contributions, and the first peak at a high binding energy value of about 532.6 eV was assigned to surface lattice oxygen in SiO_2_ (the same as that at 532.4 eV in Ni/SiO_2_). The second peak at 531.0 eV was attributed to surface adsorption oxygen (O_β_: O^2−^, O_2_^2−^, or O^−^), that was correlated to oxygen vacancy of zirconia [2,53,54]. The peak at the low binding energy of 530.0 eV was ascribed to lattice oxygen (O_α_) of ZrO_2_ [53]. As shown in Fig. 3c, by back-convolution of Zr 3d spectra, both Ni/SiO_2_@2ZrO_2_ and Ni/SiO_2_@4ZrO_2_ showed the presence of a secondary sub-oxide signal peak at 181.6 eV, which was recorded as Zr^δ+^, due to a lower oxidation state than that of the lattice Zr^4+^ (binding energy = 182.2 eV). This indicated the presence of interfacial oxygen vacancies [55]. The value of Zr^δ+^/(Zr^δ+^+Zr^4+^) and O_β_/(O_α_+O_β_) was 0.24, 0.36 and 0.35, 0.60 respectively in Ni/SiO_2_@2ZrO_2_ and Ni/SiO_2_@4ZrO_2_ by the fitted XPS data. The XPS data (Fig. S1) of the oxidized catalysts also revealed the presence of more Ni^3+^ phases over ZrO_2_ coated catalyst that provided the evidence of the formation of more oxygen vacancies, especially evident in Ni/SiO_2_@4ZrO_2_. The fitted results of the XPS spectra were recorded in Table 1. Just so you know, XPS was tested to measure the surface composition, whereas ICP-OES was performed to achieve the bulk composition of catalyst. According to XPS results, the surface Ni/Zr mole ratio for Ni/SiO_2_@2ZrO_2_ and Ni/SiO_2_@4ZrO_2_ was 0.55 and 0.20, which was far below the bulk Ni/Zr mole ratio of 1.64 and 1.32 determined by ICP-OES. This showed that the introduced ZrO_2_ was mainly coated on the surface of the catalyst.

**Fig. 4:**
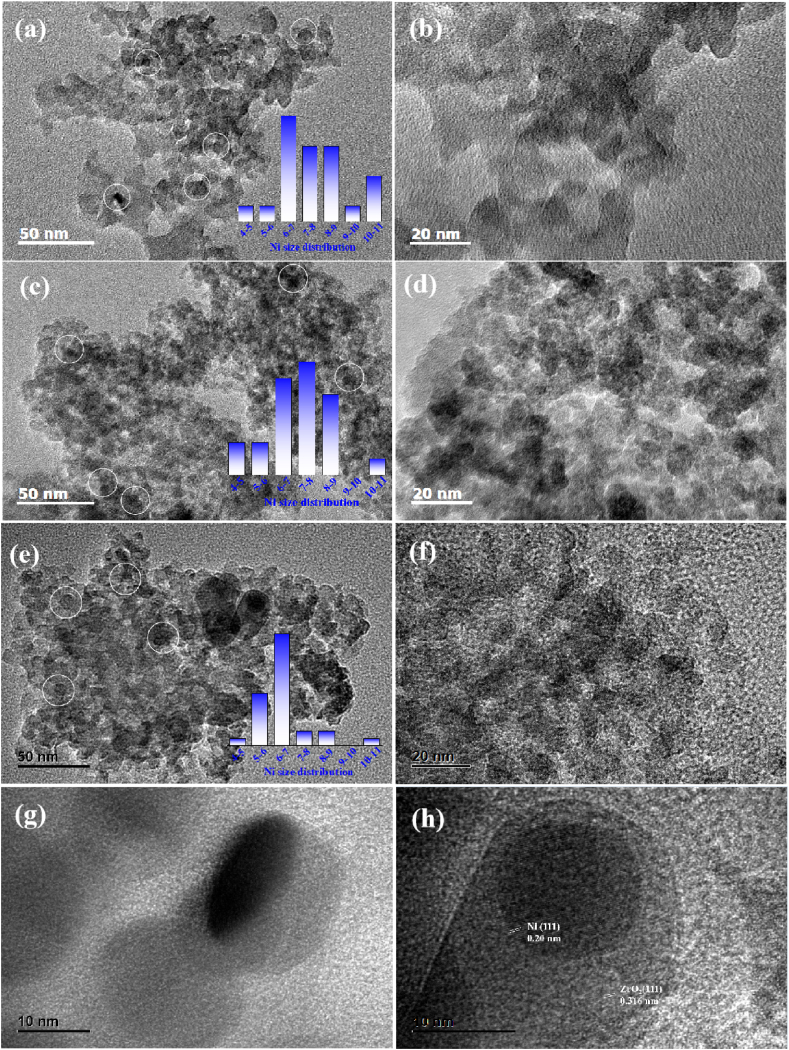
TEM images of the reduced Ni/SiO_2_ (a), Ni/SiO_2_@2ZrO_2_ (b) and Ni/SiO_2_@4ZrO_2_(c, d).

The morphology and particle dimension of the reduced Ni/SiO_2_, Ni/SiO_2_@2ZrO_2_ and Ni/SiO_2_@4ZrO_2_ catalysts were investigated by TEM and the TEM images was displayed in Fig. 4. As shown in the picture, for the three catalysts nickel particles were distributed on the support and exhibited spherical morphology. Apparently, most of the nickel particles were concentrated at 6–9 nm in the reduced Ni/SiO_2_ and Ni/SiO_2_@2ZrO_2_ catalyst, whereas that were focused on 6–7 nm in the reduced Ni/SiO_2_@4ZrO_2_ (Fig. 4a-f). This indicated the good thermal stability after calcination and reduction treatment due to the coating with ZrO_2_. The reason for the difference in Ni particle size was likely due to the influence of the reduction process. High-temperature reduction technology could inevitably accelerate metal sintering and agglomeration because of the Ostwald ripening. After coating with ZrO_2_, the ZrO_2_ shell acted as a spatial isolation role that could prevent the aggregation and sintering of nickel particles. Therefore, the average particles size of metal nickel for Ni/SiO_2_@4ZrO_2_ will be smaller than that for Ni/SiO_2_. In addition, in the reduced Ni/SiO_2_@4ZrO_2_ (Fig. 4g and h), core-shell structure that consisting of zirconia shell and Ni core was clearly presented. The HRTEM image shown in Fig. 4h further obviously visualized the lattice fringe spacing of 0.20 nm and 0.316 nm in the core-shell location, which were ascribed to the crystal lattice planes of Ni (1 1 1) and ZrO_2_ (1 1 1), respectively.

### 3.2 Catalysis in RWGS

**Fig. 5:**
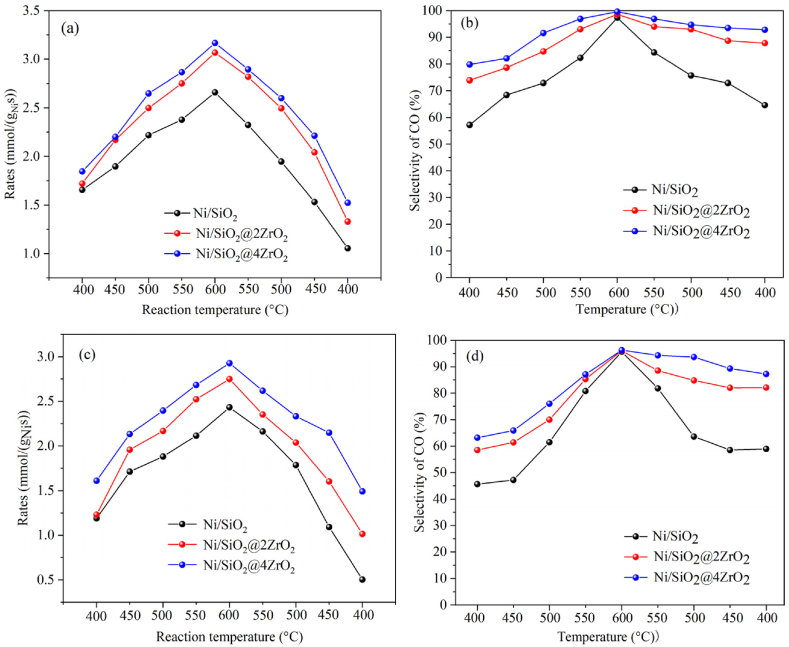
Catalytic activity of Ni/SiO_2_, Ni/SiO_2_@2ZrO_2_ and Ni/SiO_2_@4ZrO_2_ under various reaction temperatures. (a, b) GHSV = 120000mL/(g_cat_·h), (c, d) GHSV = 150000mL/(g_cat_·h).

The catalytic activity of Ni/SiO_2_, Ni/SiO_2_@2ZrO_2_ and Ni/SiO_2_@4ZrO_2_ for CO_2_ hydrogenation to CO were investigated at atmospheric pressure under the weight hourly space velocity (WHSV) of 120000 mL/(g_cat_·h) and 150000 mL/(g_cat_·h). In the CO_2_ RWGS process, besides hydrogenation product of CO and remained CO_2_, the only by-product of CH_4_ was also detected (not listed in the article). The reaction results were shown in Fig. 5. As seen in Fig. 5a, regardless of the catalysts tested, the CO_2_ conversion was closely related to the reaction temperature and gradually increased from 400 °C to 600 °C, then steadily decreased from 600 °C to 400 °C. This was consistent with the endothermic properties of the RWGS reaction. Further detailed comparison under the GHSV of 120000 mL/(gcat·h) that when the reaction temperature went from 400 °C to 600 °C, Ni/SiO_2_ exhibited increased CO_2_ conversion rates from 1.66 mmol/(g_Ni_·s) to 2.66 mmol/(g_Ni_·s), and that Ni/SiO_2_@2ZrO_2_ and Ni/SiO_2_@4ZrO_2_ from 1.72 mmol/(g_Ni_·s) to 3.07 mmol/(g_Ni_·s) and from 1.85 mmol/(g_Ni_·s) to 3.17 mmol/(g_Ni_·s), respectively. And it was clear that Ni/SiO_2_@4ZrO_2_ showed higher CO_2_ conversion rates than Ni/SiO_2_ and Ni/SiO_2_@2ZrO_2_. However, when the reaction temperature was down from 600 °C to 400 °C, the CO_2_ conversion rates of Ni/SiO_2_ were decreased from 2.66 mmol/(g_Ni_·s) to 1.05 mmol/(g_Ni_·s), that of Ni/SiO_2_@2ZrO_2_ and Ni/SiO_2_@4ZrO_2_ was decreased from 3.07 mmol/(g_Ni_·s) to 1.33 mmol/(g_Ni_·s) and from 3.17 mmol/(g_Ni_·s) to 1.53 mmol/(g_Ni_·s), respectively. It was obvious that the catalytic activity of Ni/SiO_2_ decreased substantially. It was also surprising to discover that the activity of all the three catalysts was decreased by comparing the CO_2_ conversion rate at the reaction temperature of initial 400 °C with the final 400 °C, and the rate of decline was 60.5 %, 56.7 % and 51.7 % for Ni/SiO_2_, Ni/SiO_2_@2ZrO_2_ and Ni/SiO_2_@4ZrO_2_, respectively. When the GHSV was 150000 mL/(gcat·h), this phenomenon was more remarkable. As the reaction temperature increased from 400 °C to 600 °C, the CO_2_ conversion rate of the three catalysts augmented steadily, and the catalytic activity was followed by Ni/SiO_2_@4ZrO_2_>Ni/SiO_2_@2ZrO_2_>Ni/SiO_2_. But, with the reaction temperature decreasing from 600 °C to 400 °C, the CO_2_ conversion rates of Ni/SiO_2_@2ZrO_2_ and Ni/SiO_2_@4ZrO_2_ was dropped from 2.75 mmol/(g_Ni_·s) to 1.01 mmol/(g_Ni_·s) and from 2.93 mmol/(g_Ni_·s) to 1.49 mmol/(g_Ni_·s), respectively, and that of Ni/SiO_2_ was decreased from 2.43 mmol/(g_Ni_·s) to 0.50 mmol/(g_Ni_·s). And the rate of decline was 79.2 %, 63.0 % and 48.9 % for Ni/SiO_2_, Ni/SiO_2_@2ZrO_2_ and Ni/SiO_2_@4ZrO_2_. Hence, when coating with ZrO_2_, the activity stability of the catalyst was enhanced, that was, good resistance to particle aggregation.

The results of CO selectivity under the GHSV of 120000 mL/(g_cat_·h) and 150000 mL/(g_cat_·h) for Ni/SiO_2_, Ni/SiO_2_@2ZrO_2_ and Ni/SiO_2_@4ZrO_2_ were listed in Fig. 5b and d. It was obvious that, despite of the reaction temperature, Ni/SiO_2_@2ZrO_2_ and Ni/SiO_2_@4ZrO_2_ exhibited a higher CO selectivity, particularly obvious in the low temperature range, compared with Ni/SiO_2_. This indicated that the addition of ZrO_2_ could obviously improve the CO selectivity. And when coating with ZrO_2_, there was a more steady trend of change for CO selectivity with the variation in temperature, especially for Ni/SiO_2_@4ZrO_2_. It was again proved that ZrO_2_ coated nickel-based catalyst possessed better activity stability. There was an unexpected phenomenon that the CO selectivity was higher at the reaction temperature of the final 400 °C than that of the initial 400 °C. When using GHSV of 120000 mL/(g_cat_·h), the reaction started at 400 °C with selectivity of CO as 57.2 %, 73.9 % and 79.8 %, while finished at 400 °C with selectivity of CO as 64.6 %, 87.8 % and 92.8 % for Ni/SiO_2_, Ni/SiO_2_@2ZrO_2_ and Ni/SiO_2_@4ZrO_2_. When using GHSV of 150000 mL/(g_cat_·h), this phenomenon was particularly obvious. The CO selectivity of Ni/SiO_2_, Ni/SiO_2_@2ZrO_2_ and Ni/SiO_2_@4ZrO_2_ at the initial 400 °C was 45.6 %, 58.5 % and 63.2 %, and that at the final 400 °C was 58.9 %, 82.1 % and 87.2 %. The increment rate was respectively 29.2 %, 40.3 % and 38.0 %. The similar situation was found in this report and the presence of CO_2_/H_2_ or CH_4_ atmosphere modified the structural and/or surface properties of nickel sites and suppressed further formation of methane [56].

**Fig. 6:**
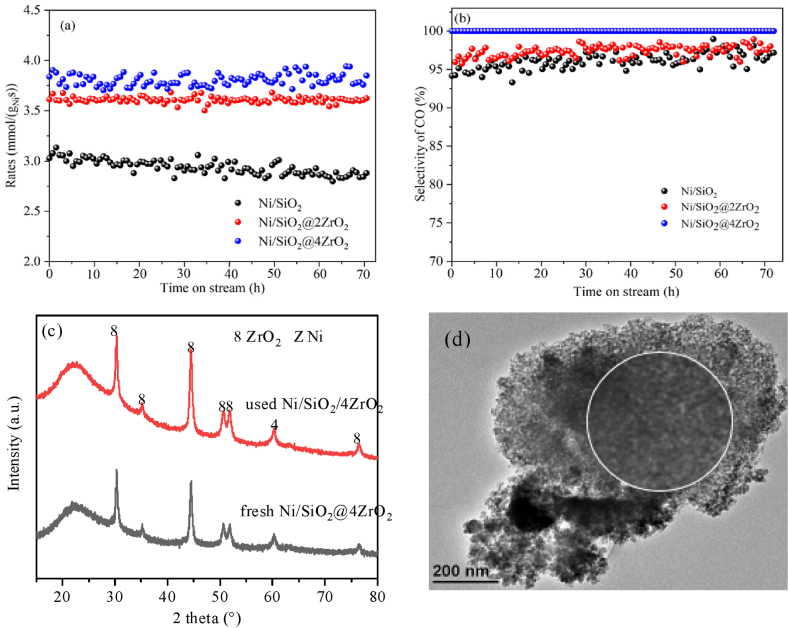
Durability test of Ni/SiO_2_, Ni/SiO_2_@2ZrO_2_ and Ni/SiO_2_@4ZrO_2_ (a, b); XRD pattern (c) and TEM image (d) of the used Ni/SiO_2_@4ZrO_2_.

The durability test of Ni/SiO_2_, Ni/SiO_2_@2ZrO_2_ and Ni/SiO_2_@4ZrO_2_ at 600 °C under the WHSV of 150000 mL/(g_cat_·h) was also measured for 72 h during CO_2_ RWGS reaction. As shown in Fig. 6a, for Ni/SiO_2_, the CO_2_ conversion rates were obviously declined from 3.03 mmol/(g_Ni_.s) to 2.88 mmol/(g_Ni_.s) after 72 h on stream. While the conversion rates of CO_2_ over Ni/SiO_2_@2ZrO_2_ and Ni/SiO_2_@4ZrO_2_ remained almost unchanged as approximately 3.60 mmol/(g_Ni_.s) and 3.80 mmol/(g_Ni_.s) at 72 h. The CO selectivity tabulated in Fig. 6b over Ni/SiO_2_ and Ni/SiO_2_@2ZrO_2_ was maintained at about 95 % and 96 %, while that over Ni/SiO_2_@4ZrO_2_ was stayed around 100 %. Besides, the XRD (Fig. 6c) of the used Ni/SiO_2_@4ZrO_2_ exhibited the characteristic diffraction peaks of Ni and ZrO_2_, with the same peak location and peak intensity to the fresh Ni/SiO_2_@4ZrO_2_. Meanwhile, the TEM images (Fig. 6d) of the used Ni/SiO_2_@4ZrO_2_ showed an excellent dispersion without evident aggregation and particle growth, suggesting a good resistance to metal sintering. This indicated that the coating of ZrO_2_ on Ni/SiO_2_ surface could achieved high activity and CO selectivity, and effectively improve the activity stability of the nickel-based catalyst.

Therefore the superior CO_2_ conversion rate and CO selectivity, as well as stability of the core-shell Ni/SiO_2_@4ZrO_2_ catalyst should be due to the facilitation of zirconia. First of all, Ni/SiO_2_@4ZrO_2_ possessed rich medium-strength CO_2_ active sites and abundant oxygen vacancies provided by ZrO_2_ that facilitated the activation and reduction of CO_2_, while SiO_2_ was merely serving as a dispersive carrier with no oxygen vacancies. For CO_2_ RWGS reaction, carbon dioxide must be firstly adsorbed efficiently on the catalyst surface (or surface oxygen vacancy) to form activated carbon species, then further reacted with atomic hydrogen to form CO and H_2_O. Therefore, it was conducive of effective active sites and oxygen vacancies to the conversion of carbon dioxide. Secondly, ZrO_2_ had hydrophilic characteristics that was conducive to the desorption of produced water, and this would definitely facilitate the RWGS reaction. Thirdly, after coating with ZrO_2_, there was a enhanced interaction between Ni and support which favored the formation of CO. Fourthly, the ZrO_2_ shell acted as a spatial isolation role that could prevent the aggregation and sintering of nickel particles. This could explain the high reaction stability of Ni/SiO_2_@4ZrO_2_.

## 4 Conclusions

This work gave a new perspective for the application of Ni/SiO_2_@ZrO_2_ for carbon dioxide hydrogenation reaction. The Ni/SiO_2_@ZrO_2_ catalyst was prepared by coating with ZrO_2_ on the Ni/SiO_2_ surface via in situ hydrothermal synthesis method. TEM results revealed that the catalyst exhibited a distinct core-shell structure with a ZrO_2_ shell, as well as uniform particle dispersion. H_2_-TPR and XPS profiles indicated the evidence of enhanced metal-support interaction and the rich oxygen vacancy in the Ni/SiO_2_@4ZrO_2_ catalyst. CO_2_-TPD studies confirmed Ni/SiO_2_@4ZrO_2_ having abundant medium-strength CO_2_ adsorption sites. These advantages endowed Ni/SiO_2_@4ZrO_2_ with superior hydrogenation activity and selectivity to CO as well as stability for the CO_2_ RWGS reaction. Therefore, the materials with core-shell structure, wherein the shell could protect metal particles from further aggregation and optimize the surface or/and electronic properties of catalyst, was expected to be further developed and utilized.

## CRediT authorship contribution statement

**Sha Cui:** Writing – original draft, Visualization, Methodology, Investigation, Funding acquisition, Formal analysis, Data curation. **Zhe Wang:** Writing – review & editing, Resources, Project administration, Investigation, Funding acquisition. **Honggang Zhao:** Writing – review & editing, Supervision, Resources, Project administration, Investigation, Funding acquisition. **Houxiang Sun:** Visualization, Project administration, Funding acquisition, Data curation. **Qinhong Wei:** Visualization, Validation, Supervision, Resources, Conceptualization. **Luhui Wang:** Visualization, Validation, Supervision, Resources, Conceptualization.

## Data availability statement

The authors confirmed that the data supporting the findings of this study were available within the article.

## Funding statement

The work was supported by the General project of 10.13039/501100008867Zhejiang Provincial Department of Education (No. Y202147639), the National Undergraduate Innovation and Entrepreneurship Training Program (No. 202210340055) and Sichuan Science and Technology Program (No. 2023NSFSC0096).

## Declaration of competing interest

The authors declare the following financial interests/personal relationships which may be considered as potential competing interests:Sha Cui reports financial support was provided by General project of 10.13039/501100008867Zhejiang Provincial Department of Education. Honggang Zhao reports financial support was provided by National Undergraduate Innovation and Entrepreneurship Training Program. Houxiang Sun reports was provided by Sichuan Science and Technology Program. If there are other authors, they declare that they have no known competing financial interests or personal relationships that could have appeared to influence the work reported in this paper.

## References

[1] Siddik M., Islam M., Zaman A.K.M.M., Hasan M.M. (2021). Current status and correlation of fossil fuels consumption and greenhouse gas emissions. Int. J. Energy Environ. Econ..

[2] Han K.H., Yu W.S., Xu L.L., Deng Z.Y., Yu H., Wang F.G. (2021). Reducing carbon deposition and enhancing reaction stability by ceria for methane dry reforming over Ni@SiO_2_@CeO_2_ catalyst. Fuel.

[3] Ding S., Liu Y. (2020). Adsorption of CO_2_ from flue gas by novel seaweed-based KOH activated porous biochars. Fuel.

[4] Kuhn A.N., Park R.C., Yu S.Y., Gao D., Zhang C., Zhang Y.H., Yang H. (2024). Valorization of carbon dioxide into C1 product via reverse water gas shift reaction using oxide-supported molybdenum carbides. Carbon Future.

[5] Zhang F., Chen W., Li W. (2023). Recent advances in the catalytic conversion of CO_2_ to chemicals and demonstration projects in China. Mol. Catal..

[6] Zheng Y.L., Liu H.C., Zhang Y.W. (2020). Engineering heterostructured nanocatalysts for CO_2_ transformation reactions: advances and perspectives. ChemSusChem.

[7] Centi G., Quadrelli E.A., Perathoner S. (2013). Catalysis for CO_2_ conversion: a key technology for rapid introduction of renewable energy in the value chain of chemical industries. Energy Environ. Sci..

[8] Sreedhar I., Varun Y., Singh S.A., Venugopal A., Reddy B.M. (2019). Developmental trends in CO_2_ methanation using various catalysts. Catal. Sci. Technol..

[9] Yang X., Su X., Chen X., Duan H., Liang B., Liu Q., Liu X., Ren Y., Huang Y., Zhang T. (2017). Promotion effects of potassium on the activity and selectivity of Pt/zeolite catalysts for reverse water gas shift reaction. Appl. Catal., B.

[10] Dong H.S., Jung M.N., Zhang Y.P., Wang S., Ding S.P. (2024). Supported noble metal-based catalysts for thermal CO_2_ hydrogenation to CO. Mol. Catal..

[11] Rabee A.I.M., Zhao D., Cisneros S., Kreyenschulte C.R., Kondratenko V., Bartling S., Kubis C., Kondratenko E.V., Bruckner A., Rabeah J. (2023). Role of interfacial oxygen vacancies in low-loaded Au-based catalysts for the low-temperature reverse water gas shift reaction. Appl. Catal., B.

[12] Li S., Xu Y., Chen Y., Li W., Lin L., Li M., Deng Y., Wang X., Ge B., Yang C., Yao S., Xie J., Li Y., Liu X., Ma D. (2017). Tuning the selectivity of catalytic carbon dioxide hydrogenation over iridium/cerium oxide catalysts with a strong metal-support interaction. Angew. Chem. Int. Ed..

[13] Choi Y., Sim G.D., Jung U., Park Y., Youn M.H., Chun D.H., Rhim G.B., Kim K.Y., Koo K.Y. (2024). Copper catalysts for CO_2_ hydrogenation to CO through reverse water–gas shift reaction for e-fuel production: fundamentals, recent advances, and prospects. Chem. Eng. J..

[14] Lu B., Kawamoto K. (2014). Transition metal-rich mesoporous silicas and their enhanced catalytic properties. Catal. Sci. Technol..

[15] Sun F.M., Yan C.F., Wang Z.D., Guo C.Q., Huang S.L. (2015). Ni/Ce–Zr–O catalyst for high CO_2_ conversion during reverse water gas shift reaction (RWGS). Int. J. Hydrogen Energy.

[16] Voigt C., Lamprecht R.E., Marushchak M.E., Lind S.E., Novakovskiy A., Aurela M., Martikainen P.J., Biasi C. (2017). Warming of subarctic tundra increases emissions of all three important greenhouse gases–carbon dioxide, methane, and nitrous oxide. Global Change Biol..

[17] Alvarez-Galvan M.C., Mota N., Ojeda M., Rojas S., Navarro R.M., Fierro J.L.G. (2011). Direct methane conversion routes to chemicals and fuels. Catal. Today.

[18] Wu H.C., Chang Y.C., Wu J.H., Lin J.H., Lin I.K., Chen C.S. (2015). Methanation of CO_2_ and reverse water gas shift reactions on Ni/SiO_2_ catalysts: the influence of particle size on selectivity and reaction pathway. Catal. Sci. Technol..

[19] Gonçalves R.V., Vono L.L.R., Wojcieszak R., Dias C.S.B., Wender H., Teixeira-Neto E., Rossi L.M. (2017). Selective hydrogenation of CO_2_ into CO on a highly dispersed nickel catalyst obtained by magnetron sputtering deposition: a step towards liquid fuels. Appl. Catal. B Environ..

[20] Zhang C., Zhang R.Y., Liu Y.X., Wu X.X., Wang H., Ge Q.F., Zhu X.L. (2023). Blocking methanation during reverse water gas shift reaction on Ni/SiO_2_ catalysts by surface Ag. ChemCatChem.

[21] Yang L., Pastor-Pérez L., Gu S., Sepúlveda-Escribano A., Reina T.R. (2018). Highly efficient Ni/CeO_2_-Al_2_O_3_ catalysts for CO_2_ upgrading via reverse water-gas shift: effect of selected transition metal promoters. Appl. Catal. B Environ..

[22] Rutherford B., Panaritis C., Pahija E., Martin C., Patarachao B., Shadbahr J., Bensebaa F., Patience G.S., Boffito D.C. (2023). Ni nanoparticles on Co_3_O_4_ catalyze the reverse water–gas shift with 95% CO selectivity at 300 °C. Fuel.

[23] Wang W.X., Li X.K., Zhang Y., Zhang R., Ge H., Bi J.C., Tang M.X. (2017). Strong metal-support interactions between Ni and ZnO particles and their effect on the methanation performance of Ni/ZnO. Catal. Sci. Technol..

[24] Wang L.H., Zhang S.X., Liu Y. (2008). Reverse water gas shift reaction over Co-precipitated Ni-CeO_2_ catalysts. J. Rare Earths.

[25] Rahmani F., Haghighi M., Estifaee P. (2014). Synthesis and characterization of Pt/Al_2_O_3_–CeO_2_ nanocatalyst used for toluene abatement from waste gas streams at low temperature: conventional vs. plasma–ultrasound hybrid synthesis methods. Microporous Mesoporous Mater..

[26] Rodrigues M.T., Zonetti P.C., Alves O.C., Sousa-Aguiar E.F., Borges L.E.P., Appel L.G. (2017). RWGS reaction employing Ni/Mg(Al,Ni)O-The role of the O vacancies. Appl. Catal., A.

[27] Wu H.C., Chang Y.C., Wu J.H., Lin J.H., Lin I.K., Chen C.S. (2015). Methanation of CO_2_ and reverse water gas shift reactions on Ni/SiO_2_ Catalysts: the influence of particle size on selectivity and reaction pathway. Catal. Sci. Technol..

[28] Liu N., Cui S., Jin Z.Y., Cao Z., Liu H., Yang S.Q., Zheng X.M., Wang L.H. (2023). Highly dispersed and stable Ni/SiO_2_ catalysts prepared by urea-assisted impregnation method for reverse water–gas shift reaction. Processes.

[29] Gioria E., Ingale P., Pohl F., Naumann d'Alnoncourt R., Thomas A., Rosowski F. (2022). Boosting the performance of Ni/Al_2_O_3_ for the reverse water gas shift reaction through formation of CuNi nanoalloys. Catal. Sci. Technol..

[30] Deng L.D., Ai X., Xie F.Q., Zhou G.L. (2021). Efficient Ni-based catalysts for low-temperature reverse water-gas shift (RWGS) reaction. Chem. Asian J..

[31] Gandara-Loe J., Zhang Q., José VilloraPicó J., Sepulveda-Escribano A., Pastor-Pérez L., Reina T.R. (2022). Design of full-temperature-range RWGS catalysts: impact of alkali promoters on Ni/CeO_2_. Energy Fuels.

[32] Zhou R., Rui N., Fan Z., Liu C. (2016). Effect of the structure of Ni/TiO_2_ catalyst on CO_2_ methanation. Int. J. Hydrogen Energy.

[33] Torres-Sempere G., González-Arias J., Penkova A., Santos-Muñoz J.L., Bobadilla L.F., Odriozola J.A., Pastor-Pérez L., Reina T.R. (2024). CO_2_ Conversion via low-temperature RWGS enabled by multicomponent catalysts: could transition metals outperform Pt?. Top. Catal..

[34] Sokolov S., Radnik J., Schneider M., Rodemerck U. (2017). Low-temperature CO_2_ reforming of methane over Ni supported on ZnAl mixed metal oxides. Int. J. Hydrogen Energy.

[35] Zonetti P.C., Letichevsky S., Gaspar A.B., Sousa-Aguiar E.F., Appel L.G. (2014). The Ni_x_Ce_0.75_Zr_0.25-x_O_2_ solid solution and the RWGS. Appl. Catal., A.

[36] Ma L.X., Ye R.P., Huang Y.Y., Reina T.R., Wang X.Y., Li C.M., Zhang X.L., Fan M.H., Zhang R.G., Liu J. (2022). Enhanced low-temperature CO_2_ methanation performance of Ni/ZrO_2_ catalysts via a phase engineering strategy. Chem. Eng. J..

[37] Zhang M., Zhang J.F., Wu Y.Q., Pan J.X. • Zhang Q.D., Tan Y.S., Han Y.Z. (2019). Insight into the effects of the oxygen species over Ni/ZrO_2_ catalyst surface on methane reforming with carbon dioxide. Appl. Catal. B Environ..

[38] Zhang D., Cai H.T., Su Y.Z., Sun W., Yang D.R., Ozin G.A. (2022). Silica samurai: aristocrat of energy and environmental catalysis. Chem Catal..

[39] Lou Y., Steib M., Zhang Q., Tiefenbacher K., Horváth A., Jentys A., Liu Y., Lercher J.A. (2017). Design of stable Ni/ZrO_2_ catalysts for dry reforming of methane. J. Catal..

[40] Gao P., Li F., Zhan H.J., Zhao N., Xiao F.K., Wei W., Zhong L.S., Wang H., Sun Y.H. (2013). Influence of Zr on the performance of Cu/Zn/Al/Zr catalysts via hydrotalcite-like precursors for CO_2_ hydrogenation to methanol. J. Catal..

[41] Guo X.M., Mao D.S., Lu G.Z., Wang S., Wu G.S. (2010). Glycine nitrate combustion synthesis of CuO-ZnO-ZrO_2_ catalysts for methanol synthesis from CO_2_ hydrogenation. J. Catal..

[42] Lim Z.Y., Wu C., Wang W.G., Choy K.L., Yin H.F. (2016). Porosity effect on ZrO_2_ hollow shells and hydrothermal stability for catalytic steam reforming of methane. J. Mater. Chem. A.

[43] Das S., Pérez-Ramírez J., Gong J.L., Dewangan N., Hidajat K., Gates B.C. • Kawi S. (2020). Core–shell structured catalysts for thermocatalytic, photocatalytic, and electrocatalytic conversion of CO_2_. Chem. Soc. Rev..

[44] Wang C.T., Guan E., Wang L., Chu X.F., Wu Z.Y., Zhang J., Yang Z.Y., Jiang Y.W., Zhang L., Meng X.J., Gates B.C., Xiao F.S. (2019). Product selectivity controlled by nanoporous environments in zeolite crystals enveloping rhodium nanoparticle catalysts for CO_2_ hydrogenation. J. Am. Chem. Soc..

[45] Dubey R.S., Rajesh Y., More M.A. (2015). Synthesis and characterization of SiO_2_ nanoparticles via sol-gel method for industrial applications. Mater. Today: Proc..

[46] Wang Y.Z., Wu R.F., Zhao Y.X. (2010). Effect of ZrO_2_ promoter on structure and catalytic activity of the Ni/SiO_2_ catalyst for CO methanation in hydrogen-rich gases. Catal. Today.

[47] Wang W.X., Li X.K., Zhang Y., Zhang R., Ge H., Bi J.C., Tang M.X. (2017). Strong metal–support interactions between Ni and ZnO particles and their effect on the methanation performance of Ni/ZnO. Catal. Sci. Technol..

[48] Li Q., Tang Q.J., Xiong P.Y., Chen D.Z., Chen J.M., Wu Z.B., Wang H.Q. (2023). Effect of palladium chemical states on CO_2_ photocatalytic reduction over g-C_3_N_4_: distinct role of single-atomic state in boosting CH_4_ production. Chin. J. Catal..

[49] Li W.Z., Zhao Z.K. (2016). Hierarchically structured tetragonal zirconia as a promising support for robust Ni based catalysts for dry reforming of methane. RSC Adv..

[50] Zhang Y., Park S.J. (2017). Au–pd bimetallic alloy nanoparticle-decorated BiPO_4_ nanorods for enhanced photocatalytic oxidation of trichloroethylene. J. Catal..

[51] Lin J.H., Ma C.P., Wang Q., Xu Y.F., Ma G.Y., Wang J., Wang H.T., Dong C.L., Zhang C.H., Ding M.Y. (2019). Enhanced low-temperature performance of CO_2_ methanation over mesoporous Ni/Al_2_O_3_-ZrO_2_ catalysts. Appl. Catal. B Environ..

[52] Jin B., Li S., Liu Y. (2022). Engineering metal-oxide interface by depositing ZrO_2_ overcoating on Ni/Al_2_O_3_ for dry reforming of methane. Chem. Eng. J..

[53] Wang H.L., Li Q., Chen J., Chen J., Jia H.P. (2023). Efficient solar‐driven CO_2_ methanation and hydrogen storage over nickel catalyst derived from metal–organic frameworks with rich oxygen vacancies. Adv. Sci..

[54] Zhou Y.D., Liu L., Li G.Y., Hu C.W. (2021). Insights into the influence of ZrO_2_ crystal structures on methyl laurate hydrogenation over Co/ZrO_2_ catalysts. ACS Catal..

[55] Ge Y.Z., Zou T.S., Martín A.J., Block T., Pottgen R., Pérez-Ramírez J. (2024). Defective zirconia promotes monometallic iron catalysts for higher alcohol synthesis. Chem Catal..

[56] Galhardo T.S., Braga A.H., Arpini B.H., Szanyi J., Goncalves R.V., Zornio B.F., Miranda C.R., Rossi L.M. (2021). Optimizing active sites for high CO selectivity during CO_2_ hydrogenation over supported nickel catalysts. J. Am. Chem. Soc..

