# Supplementary material for: Core-shell Ni/SiO2@ZrO2 catalyst for highly selective CO2 conversion accompanied by enhancing reaction stability
Source: Heliyon. 2024 Nov 27;10(23):e40697. doi: 10.1016/j.heliyon.2024.e40697 (PMC11648145; doi:10.1016/j.heliyon.2024.e40697)
Supplement: Multimedia component 1 [file mmc1.docx]

**Supporting information**

**Core-shell Ni/SiO_2_@ZrO_2_ catalyst for highly selective CO_2_ conversion accompanied by enhancing reaction stability**

Sha Cui ^a, c, d*^, Zhe Wang ^a^, Honggang Zhao ^a^, Houxiang Sun ^b^, Qinhong Wei ^a^, Luhui Wang ^a^

Figure S1 XPS profiles of (a) Ni 2p, (b) O 1s, (c) Zr 3d in oxidized Ni/SiO_2_, Ni/SiO_2_@2ZrO_2_ and [Ni/SiO](mailto:Ni/SiO2@0.8ZrO2)_[2](mailto:Ni/SiO2@0.8ZrO2)_[@4ZrO](mailto:Ni/SiO2@0.8ZrO2)_[2](mailto:Ni/SiO2@0.8ZrO2)_ catalysts.

The surface chemical state of oxidized Ni/SiO_2_, Ni/SiO_2_@2ZrO_2_ and Ni/SiO_2_@4ZrO_2_ were showed in the XPS results. In spectra of Ni 2p, all samples displayed three fitted peaks, one peak appeared at 854.1 eV with a satellite peak signal centered at 861.0 eV was assigned to Ni^2+^ in the NiO phase and another peak centered at 856.1 eV was attributed to Ni^3+^in the Ni_2_O_3_ and NiOOH^[1, 2]^. As expected, the cationic nickel species were found on the surface of all calcined catalysts. Further observation revealed that after coating with ZrO_2_, the Ni^3+^/Ni^2+^ ratio of Ni/SiO_2_@4ZrO_2_ and Ni/SiO_2_@2ZrO_2_ increased clearly comparing to Ni/SiO_2_. As verified that an increase in the proportion of Ni^3+^ at the surface indicated the formation of more oxygen vacancies^[3]^.

**References**

1. Cárdenas-Arenas A. • Bailón-García E. • Lozano-Castelló D. • Da. Costa P. •

Bueno-López A. **Stable NiO-CeO_2_ nanoparticles with improved carbon resistance for methane dry reforming.** J. Rare Earths. 2022; **40**: 57-62.

2. Long Y.X. • Zhang Z.D. • Zhao L.L. • Zeng Q.X. • Li Q. • Wang J. • Li D.Y. • Xia Q. • Liu Y. • Han X. • Zhou Z.R. • Li Y.B. • Zhang Y.M. • Chou S.L. **Bucket effect on high-performance LiO_2_ batteries based on P-doped 3D NiO microspheres with conformal growth of discharge products.** J. Mater. Chem. A. 2022; **10**: 24538-24551.

3. Zheng R.X. • Shu C.Z. • Li J.B. • Hu A.J. • Chen N. • Li M.L. • Long J.P. **Oxygen vacancy engineering of vertically aligned NiO nanosheets for effective CO_2_ reduction and capture in LiCO_2_ battery.** Electrochim. Acta. 2021; **383**: 138359.
